# Functional Implications of Plasma Membrane Condensation for T Cell Activation

**DOI:** 10.1371/journal.pone.0002262

**Published:** 2008-05-28

**Authors:** Carles Rentero, Tobias Zech, Carmel M. Quinn, Karin Engelhardt, David Williamson, Thomas Grewal, Wendy Jessup, Thomas Harder, Katharina Gaus

**Affiliations:** 1 Centre for Vascular Research, University of New South Wales and the Department of Haematology, Prince of Wales Hospital, Sydney, Australia; 2 Sir William Dunn School of Pathology, University of Oxford, Oxford, United Kingdom; 3 Faculty of Pharmacy, University of Sydney, Sydney, Australia; Ecole Normale Superieure, France

## Abstract

The T lymphocyte plasma membrane condenses at the site of activation but the functional significance of this receptor-mediated membrane reorganization is not yet known. Here we demonstrate that membrane condensation at the T cell activation sites can be inhibited by incorporation of the oxysterol 7-ketocholesterol (7KC), which is known to prevent the formation of raft-like liquid-ordered domains in model membranes. We enriched T cells with 7KC, or cholesterol as control, to assess the importance of membrane condensation for T cell activation. Upon 7KC treatment, T cell antigen receptor (TCR) triggered calcium fluxes and early tyrosine phosphorylation events appear unaltered. However, signaling complexes form less efficiently on the cell surface, fewer phosphorylated signaling proteins are retained in the plasma membrane and actin restructuring at activation sites is impaired in 7KC-enriched cells resulting in compromised downstream activation responses. Our data emphasizes lipids as an important medium for the organization at T cell activation sites and strongly indicates that membrane condensation is an important element of the T cell activation process.

## Introduction

Signals for T lymphocyte activation are transmitted at the contact zone between the T cell and a cognate antigen presenting cell (APC) [1]. The key T cell activating stimulus at the so-called immunological synapse (IS) is initiated by the T cell antigen receptor (TCR) upon binding to its cognate peptide-MHC (pMHC) ligand presented on the surface of an APC [2]. The T cell activation process is tightly coupled to spatial segregation of proteins and lipids into T cell plasma membrane domains at the IS. Segregation of these domains in the T cell plasma membrane follows several distinctive mechanisms.

Following TCR triggering, signaling protein complexes assemble in plasma membrane domains in vicinity of the TCR [3], [4]. Membrane-attached Src kinase Lck phosphorylates subunits of the TCR/CD3 complex leading to further recruitment and phosphorylation of cytosolic ZAP70 tyrosine kinase. ZAP70 phosphorylates tyrosine residues of the transmembrane protein Linker for Activation of T cells (LAT). Subsequently, LAT establishes a cooperative network of cytoplasmic signaling proteins such as the adaptor protein Grb2 and signaling enzyme PLCγ in the vicinity of activated TCR [5]. These multi-protein TCR LAT assemblies (TLAs) mediate the immediate downstream signals following TCR engagement [4] such as Ras activation [6] and induction of Ca^2+^ fluxes [7].

In addition to signaling complexes, organization of IS membrane domains is also driven by interaction of membrane bound proteins with the actin cytoskeleton. Consequently, T cells deficient in proteins which regulate and mediate actin cytoskeletal rearrangements have defects in IS formation and T cell activation [5], [8], [9].

Important steps of the T cell activation cascade have been proposed to occur in raft domains of the T cell plasma membrane. Based on studies of model membranes, lipid rafts are defined as liquid-ordered (l_o_) membrane phases coexisting with a liquid-disordered (l_d_) phase of the non-raft environment in the lipid bilayers [10]. The phase separation into l_o_/l_d_ critically depends on the presence of cholesterol. In the l_o_ phase, the planar sterol group of cholesterol is believed to align with saturated hydrocarbon chains of sphingolipids and phosphoglycerides resulting in tight lipid packing and condensation of the lipid bilayers [10], [11].

L_o_ phases in model membranes resist solubilisation by several non-ionic detergents such as Triton ×100 [12]. Thus, biochemical analysis of detergent resistant membranes (DRMs) isolated from cells was used to deduce the molecular composition of rafts. Based on these analyses, cell membrane rafts were proposed to be enriched in cholesterol, sphingolipids, and specific membrane proteins such as glycosylphosphatidyl-inositol (GPI)-anchored proteins in the outer leaflet and dual-acylated proteins anchored in the inner leaflet. However due to numerous ambiguities of detergent treatment, significant concerns were raised as to the extent to which DRMs represent domains of intact cell membranes [13], [14].

The involvement of membrane rafts as signaling platforms at the T cell activation sites was initially proposed based on the association of several membrane-associated TCR signaling proteins with DRMs [15], including acylated Src-related tyrosine kinases Lck and Fyn, acylated transmembrane linkers and TCR components. However, microscopy studies of intact T cells revealed no coclustering of generic DRM-associated raft markers such as GPI-anchored raft reporter proteins with activated TCR [16]. In contrast, the membrane polarity reporter Laurdan revealed unequivocally the formation of condensed plasma membrane domains at T cell activation sites [17] demonstrating physical hallmarks of rafts at these membrane regions.

The functional role of raft domains in T cell activation has been previously examined by disrupting ordered membrane phases by depletion of endogenous cholesterol using methyl-β-cyclodextrin (mβCD) [18]–[20] or cholesterol oxidase [19], [20]. In line with the cholesterol dependence of l_o_ phase formation, mβCD extraction reduces the accumulation of condensed raft domains at T cell activation sites [17] and causes the loss of DRM association of Lck and LAT [18]. MβCD-mediated cholesterol extraction also resulted in the inhibition of a key early T cell activation response-the induction Ca^2+^ fluxes in response to TCR triggering. Further analyses showed that mβCD extraction led to depletion of Ca^2+^ from intracellular stores which are essential for T cell activation [20]. Thus manipulation of cholesterol can disrupt several T cell functions, which makes it difficult to draw conclusions and the functional role of plasma membrane condensation in T cell activation responses remains a fundamentally important, yet unanswered question. Here, we specifically inhibit membrane condensation at T-cell activation sites using the oxysterol 7-ketocholesterol (7KC), which inhibits tight packing of saturated acyl chains and monitor the effects of 7KC on T cell signaling and early activation responses.

## Results

### Inhibition of membrane condensation at T cell activation sites by 7-ketocholesterol

The T cell plasma membrane bilayer condenses at the site of activation within minutes post stimulation [17]. In order to specifically interfere with this condensation we incorporated 7-ketocholesterol (7KC) into naïve T cells and T cell lines. 7KC differs from cholesterol only by an additional ketone group, which protrudes perpendicular from the cholesterol ring. While cellular processing and intracellular trafficking of 7KC is identical to cholesterol [21], substitution of 7KC for cholesterol decreases lipid order and increases bilayer polarity [22].

We incubated Jurkat-derived T cell lines or primary T cells with identical amounts of sterols (58 μM)-either cholesterol (CH) or 7KC alone, or as mixtures of CH and 7KC at 2:1 or 1:2 molar ratios. Sterols were incubated with the cells as water-soluble complexes with methyl-β-cyclodextrin (mβCD). The mβCD concentration in these experiments was 0.5 mM which is ∼2–20-fold lower than the concentration generally used for extraction of endogenous cholesterol from cells. Control cells were neither incubated with sterols nor with mßCD. In both Jurkat-derived T cell lines, sterol treatment resulted in a 1.4–2.0-fold increase of total cellular sterol levels (Table 1A). Upon sterol enrichment, similar cholesterol levels were measured in all treatment conditions in Jurkat 8.2 cells (38.3±8.1 nmol/mg protein) and in JCaM2 wt LAT cells (35.0±7.6 nmol/mg protein). 7KC levels ranged from 0–68% of total sterol (0–40 nmol/mg protein in Jurkat 8.2 cells; 0–38 nmol/mg protein in JCaM2 wt LAT cells).

**Table 1:** Effect of sterol treatment on membrane composition and structure.

A. Sterol composition				
	JCaM2 cells		Jurkat 8.2	
Treatments	CH+7KC nmol/mg	7KC (%)	CH+7KC nmol/mg	7KC (%)
Control	30.78±4.92	Not detect.	35.65±1.61	Not detect.
CH	42.90±4.75	Not detect.	48.90±3.87	Not detect.
2∶1 CH:7KC	52.94±9.86	20.7±1.38	54.95±4.07	20.60±1.24
1∶2 CH:7KC	58.89±9.75	41.0±4.43	57.85±1.46	39.69±3.23
7KC	62.26±5.11	68.4±5.53	53.72±0.41	58.72±3.36
**B. GP values at TCR activation and control sites**				
**JCaM2 wt LAT, 7 min**	**Anti CD3 Ab beads**		**Anti TfR Ab beads**	
Control	0.437±0.054 (86)^**^		0.220±0.076 (78)	
CH	0.437±0.063 (76)^**^		0.227±0.098 (62)	
2∶1 CH:7KC	0.325±0.080 (78)^**^		0.215±0.087 (60)	
1∶2 CH:7KC	0.247±0.078 (75)		0.228±0.091 (61)	
7KC	0.130±0.078 (80)		0.123±0.069 (60)	
**Jurkat 8.2, 7min**	**With Antigen**		**Without Antigen**	
Control	0.425±0.061 (37)^**^		0.201±0.066 (36)	
CH	0.454±0.045 (37)^**^		0.210±0.072 (34)	
2∶1 CH:7KC	0.306±0.066 (39)^**^		0.207±0.075 (33)	
1∶2 CH:7KC	0.262±0.079 (39)^*^		0.216±0.064 (33)	
7KC	0.221±0.086 (38)		0.216±0.072 (36)	
**Jurkat 8.2, 23min**	**With Antigen**		**Without Antigen**	
Control	0.342±0.073 (44)^**^		0.223±0.073 (38)	
CH	0.337±0.079 (39)^**^		0.200±0.073 (33)	
2∶1 CH:7KC	0.276±0.073 (39)^**^		0.206±0.075 (39)	
1∶2 CH:7KC	0.235±0.078 (41)		0.203±0.080 (31)	
7KC	0.181±0.082 (45)		0.186±0.059 (35)	
**C. Mean GP (and percentage coverage) of fluid and ordered populations**				
**JCaM2 wt LAT, 7min**	**Anti CD3 Ab beads**		**Anti TfR Ab beads**	
	Fluid/Ordered populations		Fluid/Ordered population	
Control	0.239 (77.7%)	0.400 (22.3%)	0.245 (89.6%)	0.409 (10.3%)
CH	0.246 (76.5%)	0.422 (23.5%)	0.251 (88.6%)	0.429 (11.4%)
2∶1 CH:7KC	0.239 (83.2%)	0.402 (16.8%)	0.250 (88.6%)	0.394 (11.4%)
1∶2 CH:7KC	0.241 (86.4%)	0.393 (13.6%)	0.239 (88.7%)	0.385 (11.3%)
7KC	0.238 (86.4%)	0.382 (11.5%)	0.178 (90.6%)	0.375 (9.4%)

**A.** Mean±standard deviation (3 experiments) of total sterol contents (CH+7KC) of JCaM2 and Jurkat 8.2 cells are given in nmol/mg cell protein. 7KC levels are a percentage of total sterol levels. **B**. Mean±standard deviation (of n images) of GP values at contact sites between JCaM2 cells and beads after 7 min of activation and between Jurkat 8.2 cells and B cells in the presence or absence of antigen after 7 min and 23 min of activation. Statistically significant differences between activation and control sites are indicated with ^**^ P<0.001, ^*^ P<0.05. **C.** Global GP values of JCaM2 cells after 7 min of activation. Normalized GP distributions were fitting to two Gaussian populations (fluid and ordered). The mean GP value (and coverage) of each population is given.

**Figure 1:**
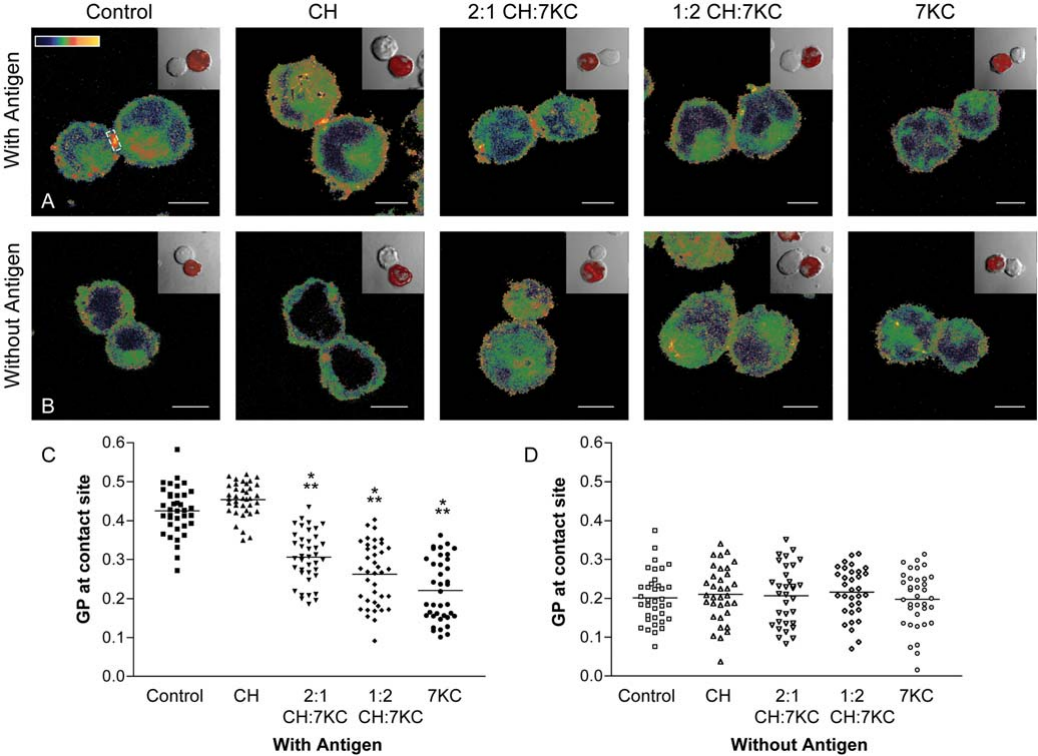
Membrane structure at immunological synapses in sterol-enriched T cells. Jurkat 8.2 T cells were treated without sterols (control), cholesterol (CH), 2:1 CH:7KC, 1:2 CH:7KC or 7KC alone as described in *Methods*. Sterol-enriched, Laurdan-labeled Jurkat 8.2 cells were conjugated to CMRA-labeled B cells for 7 min in the presence (with antigen, A, C) or absence (without antigen, B, D) of 2 μM antigen cytochrome c. A–B. Cell couples were fixed, imaged and intensity image converted into GP images and pseudo-coloured (scale in A) as described previously. Inserts show the corresponding transmission image with the orange fluorescence of CMRA overlaid to identify APCs. Bar 5 μm. C–D. GP values were measured over the entire contact area between T cell-APC couples as indicated in A. Means (indicated by horizontal lines) and SDs are shown in Table 1. In C, differences (P<0.05) were found between the means of all conditions except between control *versus* cholesterol and 1:2 CH:7KC *versus* 7KC. No differences were found in D. Difference of P<0.05 compared to control cells are marked with one asterisk; differences of P<0.05 to cholesterol-enriched cells with two asterisks.

**Figure 2:**
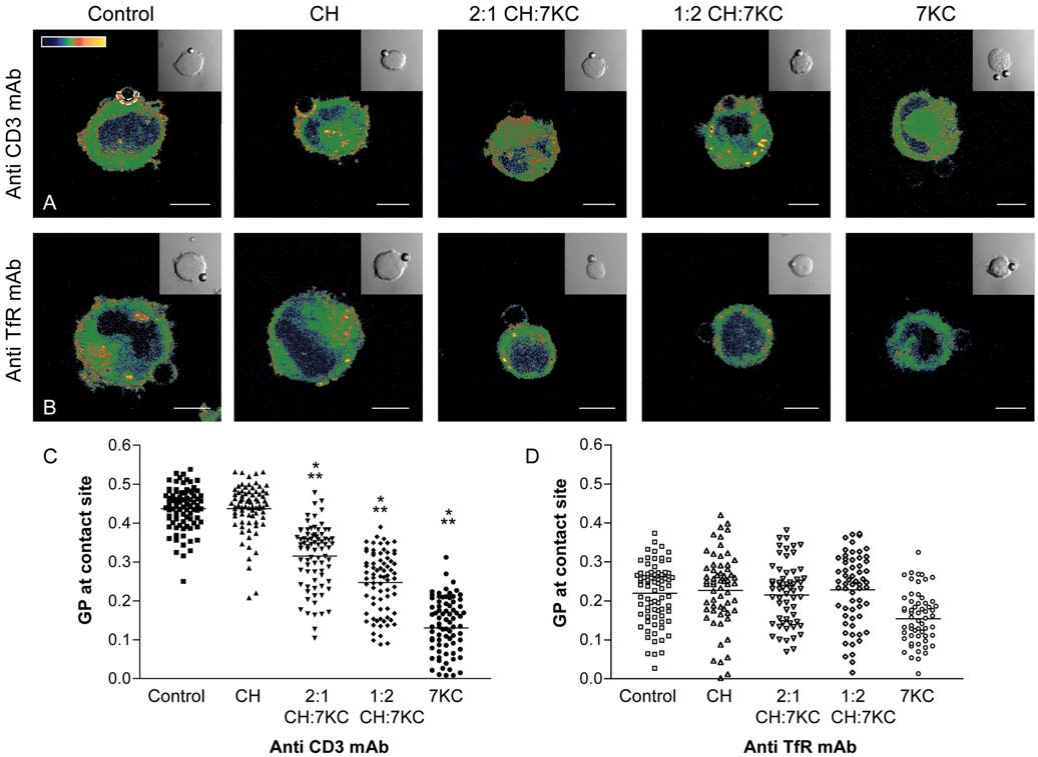
Membrane structure at activation sites in sterol-enriched T cells. Sterol-enriched JCaM2 T cells were conjugated to polystyrene beads coated with anti-CD3 mAb (Anti CD3 mAb, A, C) or anti-transferrin receptor (Anti TfR mAb, B, D) for 7 min. A–B. Cell-bead conjugates were fixed, imaged and intensity image converted into GP images and pseudo-coloured (scale in A) as described previously. Inserts show the corresponding transmission image to identify the location of the bead. Bar 5 μm. C–D. GP values were measured over the entire contact area between cells and beads. Means (indicated by horizontal lines) and SDs are shown in Table 1. In C, differences (P<0.001) were found between the means of all conditions except between control *versus* cholesterol. No differences were found in D except between 7KC-treated cells and all other conditions (P<0.05). Difference of P<0.05 compared to control cells are marked with one asterisk; differences of P<0.05 to cholesterol-enriched cells with two asterisks.

Sterol-treated Jurkat 8.2 cells, which express the 5C.C7 TCR reactive to the moth cytochrome C (MCC) peptide 87–103 in the context of IEk class II MHC, were conjugated with±MCC peptide-pulsed CH27 IEk expressing B-cells (Fig. 1). JCaM2 wt LAT cells, which are fully TCR responsive, were conjugated to polystyrene beads coated with TCR-activating anti-CD3 monoclonal antibodies (mAb) or with anti-transferrin receptor (TfR) mAb coated control beads which do not activate T cells (Fig. 2).

Plasma membrane structure at the respective T cell activation sites was visualized (Fig. 1A, B; Fig. 2A, B) and quantified (Fig. 1C, D; Fig. 2C, D) as described previously [17] using the fluorescent membrane dye Laurdan (5 μM labeling concentration) and 2-photon microscopy. Laurdan shifts its peak emission wavelength from ∼500 nm in fluid to ∼440 nm in ordered membranes [23]. Thus the normalized ratio of two, simultaneously recorded, intensity channels, defined as the generalized polarization (GP), is a relative measure of membrane order with fluid domains in cellular membranes yielding typically a GP value below 0.3 [24].

To demonstrate that interactions with TCR membrane proteins are not solely responsible for the spectral properties of the probe, we conducted fluorescence resonance energy transfer (FRET) studies in activated T cells using tryptophan fluorescence as a donor for Laurdan excitation [25]. At low Laurdan concentrations in the labeling media (<10 μM), Laurdan was not in the vicinity of tryptophan-containing proteins to result in detectable FRET. High levels of the probe resulted in FRET (Fig. S1A) concomitant with a small increase in mean GP value at the activation site (Fig. S1B). The high concentrations of Laurdan required to achieve FRET indicate that the probe does not specifically interact with proteins. Further Laurdan within the vicinity of proteins reports similar GP values than outside the FRET range (estimated to be 14 Å in liposomes [25]) with a variation in mean GP of±0.04. Thus the probe is likely to report the overall membrane structure of a defined region.

GP values were pseudo-colored in cell images, as indicated in Figs. 1A and 2A. We measured the contact site between MCC peptide-pulsed *versus* not pulsed CH27 B-cells with Jurkat 8.2 cells (Fig. 1) and between JCaM2 wt LAT cells and beads coated with monoclonal antibodies (mAb) to CD3 *versus* transferrin receptor (TfR, Fig. 2) to compare GP values at activation with those of non-activation control sites. As previously reported [17], we observed a significant condensation at the site of Jurkat 8.2 cell activation by B cells pulsed with antigenic peptide (Fig. 1A, Table 1B) and the site of JCaM2 wt LAT cell contacts with anti-CD3 coated beads (Fig. 2A, Table 1B). Cholesterol-only treatment did not affect this activation-induced increase in membrane condensation, but 7KC significantly reduced mean GP values and hence membrane condensation at the T cell activation sites in a dose dependent manner (Fig. 1 and Fig. 2; Table 1).

Under most 7KC loading conditions, we observed little effect on the order of the plasma membrane outside T cell activation sites. However, at the highest 7KC dose used, we observed a reduction of the mean GP value (from ∼0.22 to 0.123) at the sites of conjugation of JCaM2 wt LAT cells with anti-TfR coated beads (Fig. 2D). To further evaluate the global effects of 7KC on membrane density, we deconvoluted the GP distribution of the cell images to discriminate fluid and ordered membrane populations (mean GP and coverage in parentheses, Table 1C), as described earlier [17], [24]. With increasing doses of 7KC in CD3-activated JCaM2 wt LAT cells, the proportion of the ordered membrane population decreased from 23% to 11% while the mean GP value of this population did not change significantly. Non-stimulated cells exhibited no significant decrease in the ordered membrane population in response to 7KC (from 10.3% to 9.4%). Thus we established conditions at which 7KC appears to specifically impair membrane condensation at T cell activation sites while bulk membranes and control, non-activation sites are not affected.

### Effects of sterol enrichment on cell viability and protein expression

**Figure 3:**
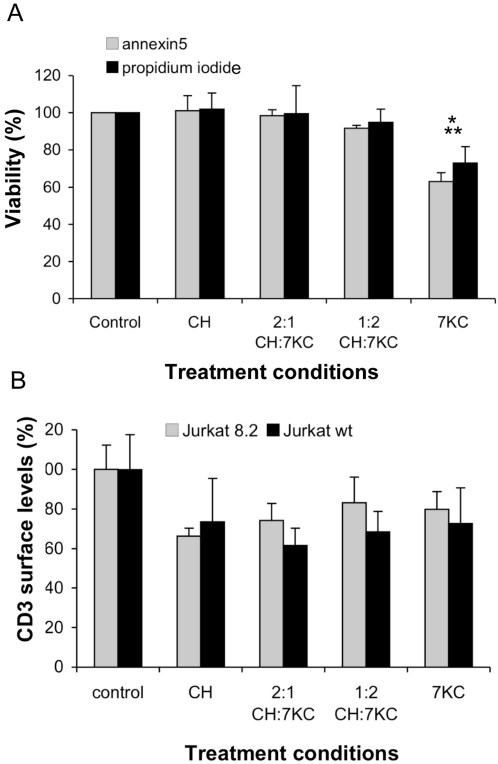
Viability and TCR surface expression sterol-enriched T cells. A. Viability of sterol-treated Jurkat cells was determined with annexin-5 staining to detect apoptosis and propidium iodide for necrotic cells. Viability was normalized to control cells. One asterisk denotes P<0.001 compared to control; two asterisks indicate P<0.05 compared to cholesterol-enriched cells. B. Surface expression of CD3 was determined by flow cytometry in wild-type Jurkat and Jurkat 8.2 cells and normalized to control cells.

It has been previously reported that high levels of 7KC induces apoptosis by activating caspases [26], [27] and generating reactive oxygen species (ROS) [28]. We performed a series of experiments to exclude the possibility that 7KC treatment had other effects, which may be responsible for reduced T cell activation responses. First, we tested whether 7KC caused cell death (Fig. 3A) as high levels of 7KC have been previously reported to be cytotoxic in some cells [29]. Indeed, we did observe increased cell death at the highest 7KC loading (P<0.05) compared to control or cholesterol-enriched cells but enrichment with lower 7KC concentrations with 2:1 CH:7KC or 2:1 CH:7KC had no significant effect (P>0.05) on cell viability. Similarly, only cells enriched with the highest 7KC concentration displayed a change in mitochondrial potential (Fig. S2A), while lower concentrations had no significant effect. ATP levels were decreased in all sterol loading conditions with no differences between cholesterol and 7KC enrichment (Fig. S2B). Further, we found no differences between control and sterol treatment conditions in caspase-3 activation (Fig. S2C), ROS levels (Fig. S2D) and cell size (data not shown). In summary, toxic effects of 7KC are unlikely to explain the impaired TCR signaling and activation responses in T cells enriched with 20% 7KC (treated with 1:2 CH:7KC) or ∼40% 7KC (1:2 CH:7KC).

We also examined the expression of key signaling proteins (Fig. S2E) and sterol-sensitive genes (Fig. S2F) in control, cholesterol- and 7KC-enriched cells, with no significant differences in expression between all five conditions. However, surface expression of TCR is reduced by 20–35% in all sterol-loaded Jurkat cells but we found no difference between cholesterol-only and 7KC-treated cells (Fig. 3B). In the following assays, we thus analyze T cell responses of 7KC-enriched cells relative to cholesterol-enriched cells to compare cells with similar TCR surface expression. In summary, the correlation between membrane structure and signal transduction is unlikely to be a consequence of increased T cell death or altered TCR signaling protein expression.

### Early T cell signaling activities in sterol-enriched T cells

**Figure 4:**
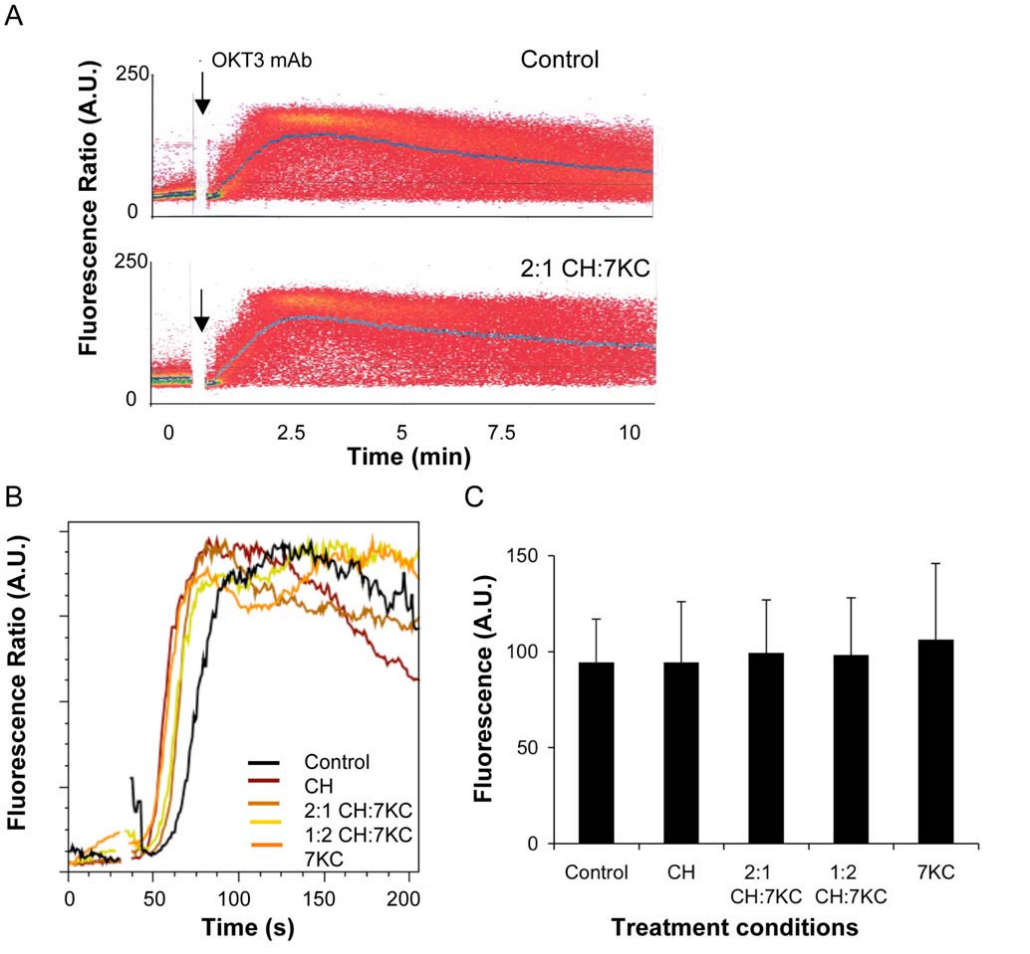
Calcium flux upon TCR triggering. A. Jurkat cells (untreated controls or treated with 2:1 CH:7KC) were loaded with Indo-1 and evaluated for Ca^2+^ mobilization by stimulation with 5 μg OKT-3 (anti-CD3 mAb) after 60 s (see arrow). The mean Ca^2+^ flux is indicated by the blue lines. B. Mean calcium mobilization upon TCR triggering for the indicated sterol conditions. C. Maximum fluorescence intensity of Fluo-4-labeled Jurkat cells (15–40 cells per condition) stimulated anti-CD3-mAb adhered to microscopy coverslips. No differences between cell conditions were found (P>0.05).

Cholesterol depletion with mßCD has been shown to inhibit intracellular Ca^2+^ fluxes independent of membrane order (Pizzo et al, 2002). We next tested the Ca^2+^ fluxes elicited by soluble OKT3 mAb-mediated TCR triggering in 7KC-enriched Jurkat cells. FACS analyses of Jurkat cells labeled with Indo-1 fluorescent Ca^2+^ sensor (Fig. 4A) revealed no differences in mean Ca^2+^ fluxes between control, cholesterol or 7KC-enriched T cells (Fig. 4B). To measure Ca^2+^ fluxes when T cells are activated locally, sterol-enriched cells were allowed to settle on anti-CD3 antibody-coated coverslips on an inverted microscope. As soon as the cells made contact with the activating surface, the fluorescence intensity of the calcium indicator Fluo-4 was recorded. When cells were thus activated with adhered antibodies, we also found no difference in peak fluorescence corresponding to intracellular calcium concentration (Fig. 4C) or response time (data not shown). Hence, we established conditions that inhibit membrane condensation but do not affect TCR-mediated Ca^2+^ fluxes. Thus tyrosine kinases, adaptor proteins and signaling enzyme PLCγ function sufficiently to induce Ca^2+^ fluxes upon TCR triggering.

**Figure 5:**
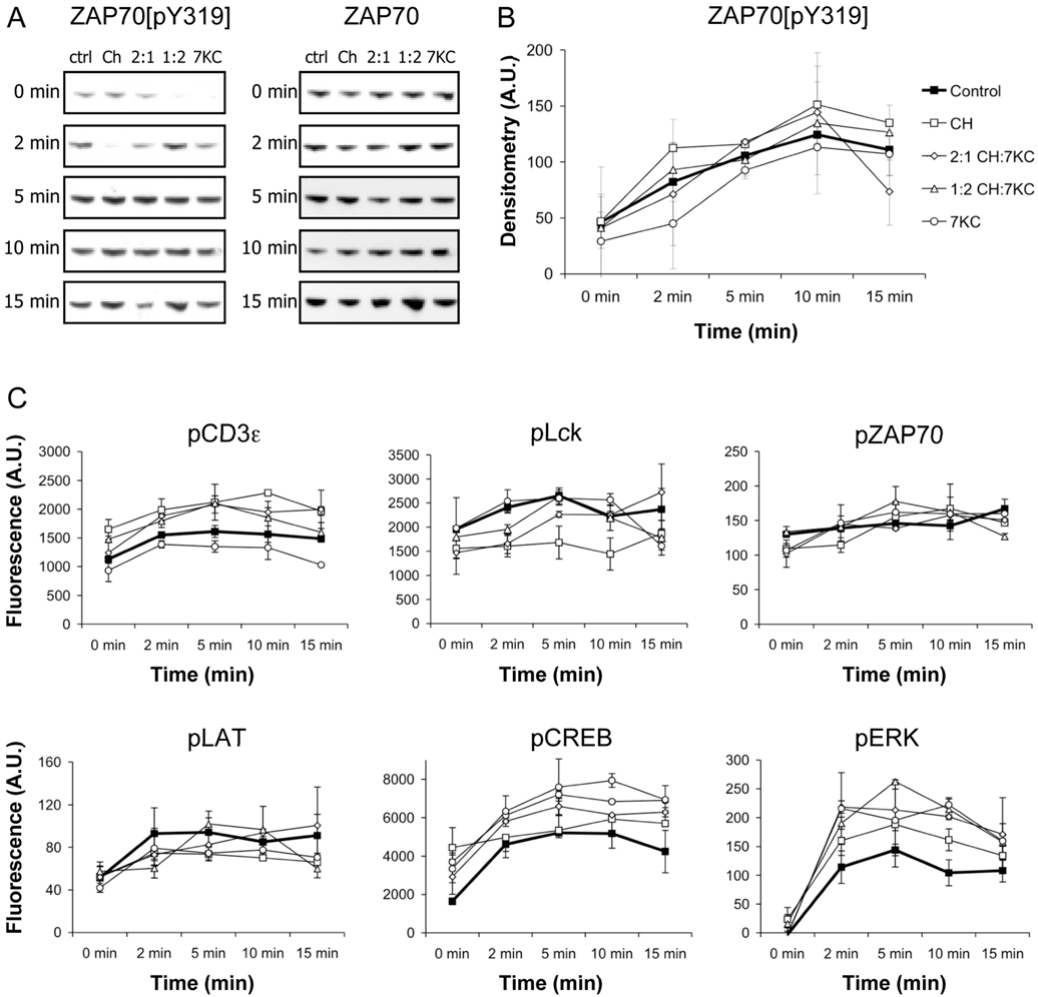
Signaling activities upon TCR triggering in whole cell lysates. A. Control and sterol-enriched Jurkat cells were activated with 5 μg of UCHT1 (anti-CD3 mAb) for the indicated periods of time. Whole cell lysates were probed for ZAP70 phosphorylated at tyrosine 319. B. Quantification of tyrosine 319 phosphorylation of ZAP70. The data show the mean and range of two independent experiments. C. Multiplex analysis of T cell signaling. 2×10^6^ sterol-enriched Jurkat wt cells were activated with 5 μg/ml of anti-CD3 UCHT1 antibody for 0-15 min at 37°C. Non-site specific tyrosine phosphorylation of CD3ε, Lck, ZAP70, LAT and ERK1/2 (Tyr185/Tyr187) as well as serine phosphorylation of CREB (Ser133) in whole cell lysates was assessed by multiplex microbead suspension assay. The data is one representative experiment; error bars represent standard deviations. Legend shown in B applies to data in C.

We further tested early signaling events upon TCR triggering in whole cell lysates by probing for tyrosine phosphorylation with immunoblotting (Fig. 5A and B) or multiplex analysis using a microbead suspension assay (Fig. 5C). Surprisingly, we found no differences in the degree or rate of specific (Fig. 5B) or total tyrosine phosphorylation of CD3ζ, Lck, ZAP70, LAT, ERK and CREB (Fig. 5C). We also found no differences between the five cell conditions in PLCγ1 phosphorylation upon TCR stimulation. Differences within an assay such as the lower phosphorylation of Lck in cholesterol-enriched T cells were not reproduced in independent experiments. It is possible that the assays employed here, particularly immunoblotting, are not sensitive enough to detect small changes in overall phosphorylation rate but it appears that signaling activities in whole cell lysates via tyrosine phosphorylation are unaltered in sterol-enriched T cells.

### Inhibition of T cells membrane condensation impairs assembly of TCR signaling clusters on the cell surface

Multi-molecular signaling assemblies form upon TCR triggering in T lymphocyte plasma membranes. For detergent-free biochemical characterization of these assemblies, we immunoisolated TCR signaling domains from sterol-loaded Jurkat cells and analyzed them by immunoblotting as established previously [30]. Briefly, Jurkat T cells were conjugated on ice to magnetic beads coated with TCR triggering anti-CD3 mAb, then activated by incubation at 37°C. Subsequently the conjugates were homogenized mechanically by nitrogen cavitation. Plasma membrane fragments containing the TCR signaling domains bound to the magnetic beads were retrieved with a magnet and subjected to immunoblotting.

**Figure 6:**
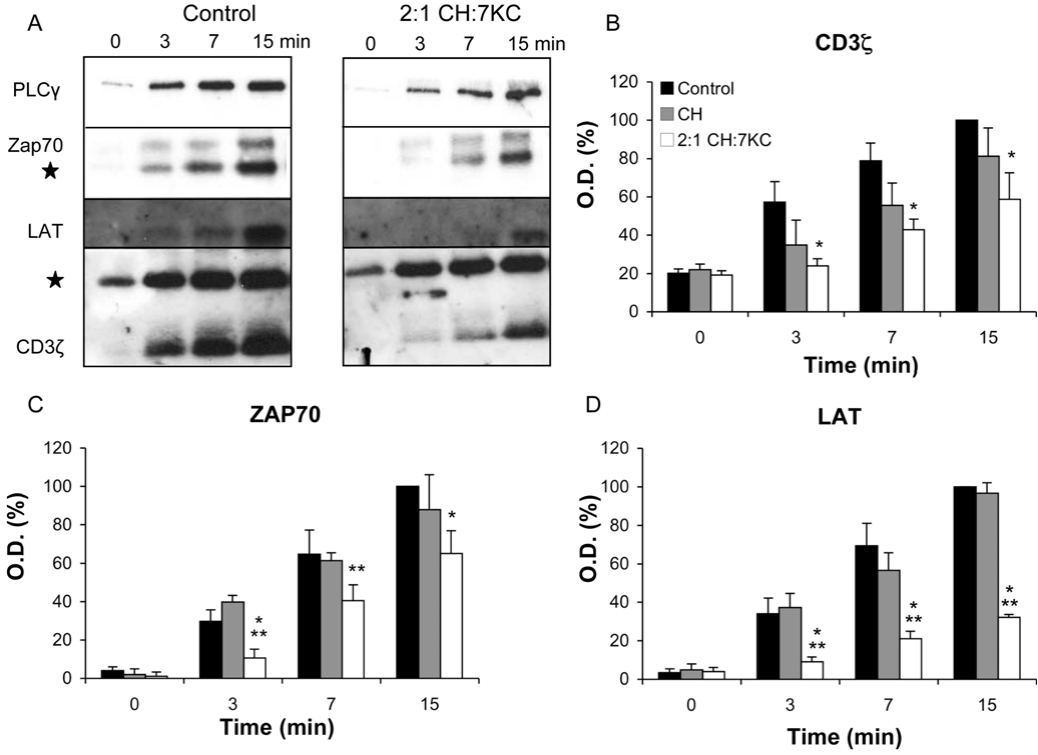
Formation of signaling complexes in sterol-enriched T cells. A. Sterol-treated Jurkat T cells (A control and 2:1 CH:7KC) were conjugated with magnetic beads coated with anti-CD3 monoclonal antibodies. Cell-bead conjugates were activated for 0–15 min at 37°C and subsequently homogenized by nitrogen cavitation at 4°C. Proteins from the recovered beads were separated by SDS page electrophoresis and probed for signaling proteins CD3ζ, ZAP70, LAT and PLCγ. Detection of proteins indicates recruitment to the activation site. The star denotes the antibody heavy chain. B–D. Quantification of the recruitment of CD3ζ (B), ZAP70 (C) and LAT (D). One asterisk indicates a significant difference compared to control cells (P<0.05); two asterisks indicate a significant difference compared to cholesterol-enriched cells (P<0.05).

We probed isolated signaling complexes for CD3ζ, ZAP70, LAT and PLCγ (Fig. 6A). As described previously [30], activation of non-sterol treated (control) cells resulted in formation of TCR-LAT signaling assemblies with a strong and sustained recruitment. In 7KC-enriched cells (2:1 CH:7KC), recruitment of CD3ζ, ZAP70, LAT and PLCγ is clearly less efficient with a delayed assembly of the signaling complexes, while this is not the case in cholesterol-enriched cells (Fig. S3A). We quantified the recruitment of CD3ζ (Fig. 6B), ZAP70 (Fig. 6C) and LAT (Fig. 6D) isolated from control, cholesterol and 7KC-enriched (2:1 CH:7KC) cells. The similar levels of antibody heavy chain recovered in the immuno-isolated membrane fragments indicates equal recovery of beads post cell homogenation (Fig. S3B). In sterol-loaded cells, recruitment of CD3ζ to the anti-CD3 isolates was only reduced in comparison to control cells, which may be caused by the reduced TCR surface expression (Fig. 3B). In cholesterol-loaded cells, neither the recruitment of CD3ζ nor of ZAP70 and LAT to the anti-CD3 immunoisolates exhibits significant difference to control cells. Only 7KC enrichment resulted in a consistent and significant reduction of ZAP70 and LAT to the anti-CD3 isolates compared to cholesterol-loaded cells. This effect was particularly striking in the amount of LAT that was found in the anti-CD3 isolates. In summary, we observed that 7KC reduced the formation of TCR signaling assemblies.

**Figure 7:**
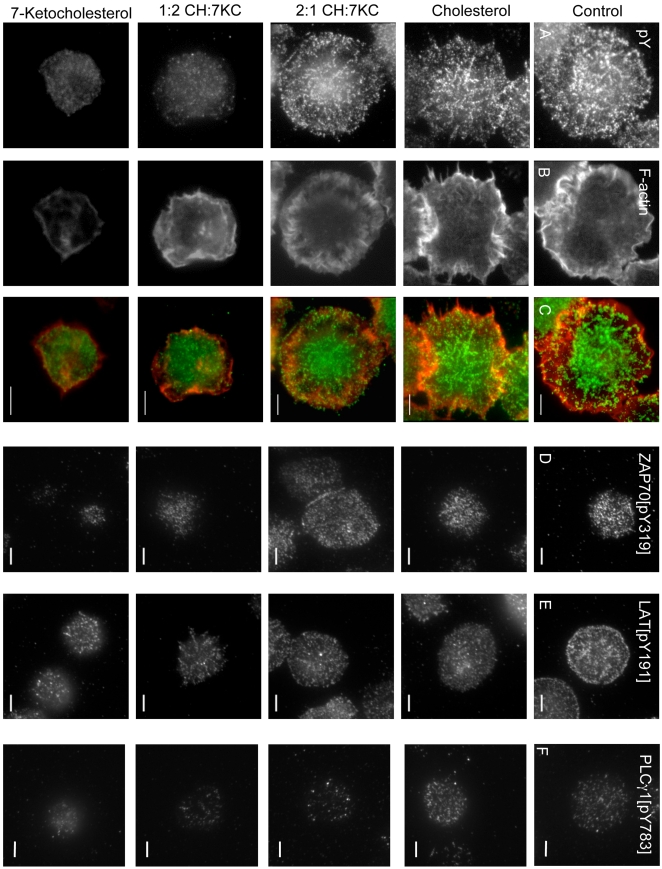
TIRF microscopy images of TCR activation sites of sterol-enriched T cells. Sterol-treated Jurkat cells were activated for 10 min on anti-CD3 mAb-coated glass coverslips, fixed and stained for phosphotyrosine (pY, A), phalloidin (B), ZAP70 phosphorylated at tyrosine 319 (D), LAT phosphorylated at tyrosine 191 (E) or PLCγ1 phosphorylation at tyrosine 783 (F). T cell activation sites were imaged by TIRF microscopy with a penetration depth of ∼100 nm. Panel C show the merged images with pY in green and F-actin in red. Bar 5 μm.

We independently monitored the formation of TCR activation clusters using total internal reflection fluorescence (TIRF) microscopy in sterol-treated Jurkat T cells (Fig. 7). The cells were allowed to settle on anti-CD3 mAb coated coverslips for 10 min at 37°C, fixed and probed for total tyrosine phosphorylation (pY, Fig. 7A and colored green in Fig. 7C), stained with phalloidin for F-actin (Fig. 7B and colored red in Fig. 7C), probed for ZAP70 phosphorylated at tyrosine 319 (Fig. 7D), LAT phosphorylated at tyrosine 191 (Fig. 7E) or PLCγ1 phosphorylated at tyrosine 783 (Fig. 7F). In control and cholesterol-loaded cells, a large number of tyrosine phosphorylation spots were visible at the activation site surrounded by a ring of F-actin as described previously [31], [32]. With increasing levels of 7KC, activation sites were smaller, tyrosine phosphorylation positive spots reduced in number and brightness (quantification in Fig. S4A) and the F-actin ring became less pronounced (Fig. S4B) with some small and bright F-actin spots visible in 7KC-enriched cells. Jurkat cells plated on poly-L-lysine- or transferrin receptor (TfR)-coated coverslips, under all sterol treatments spread and formed similarly bright phalloidin-stained lamellipodia (data not shown) indicating that 7KC-enriched cells are capable of actin polymerisation but are specifically deficient in producing TCR signals to mediate formation of actin rings. The intensity of phospho-ZAP70 staining was similar in control cells and cells enriched with cholesterol or 2:1 CH:7KC but reduced significantly at higher doses of 7KC (Fig. S4C). In contrast, phospho-LAT was significantly reduced in all sterol conditions (Fig. S4D) correlating with the reduced TCR surface expression (Fig. 3B). Importantly, as in immuno-isolated TCR activation sites, 7KC enrichment reduced phospho-LAT at the cell surface in a dose-dependent manner and to a greater extent than cholesterol enrichment. Similarly, PLCγ1 was significantly lower in 7KC-enriched cells compared to cholesterol-enriched or control cells so that the degree of pLAT correlate well with pPLCγ1. In summary, the microscopy data of reduced tyrosine phosphorylated proteins at the cell surface agrees extremely well with our biochemical observations of diminished signaling complexes in the plasma membrane of 7KC-enriched cells. Taken together, our data suggests that 7KC modulates the location of TCR signaling proteins with less signaling proteins recruited or retain at the cell surface and this impacts more severely on LAT or actin restructuring than on upstream components such as ZAP70 or elements of the CD3 complex.

### Downstream activation responses of sterol-enriched T cells

**Figure 8:**
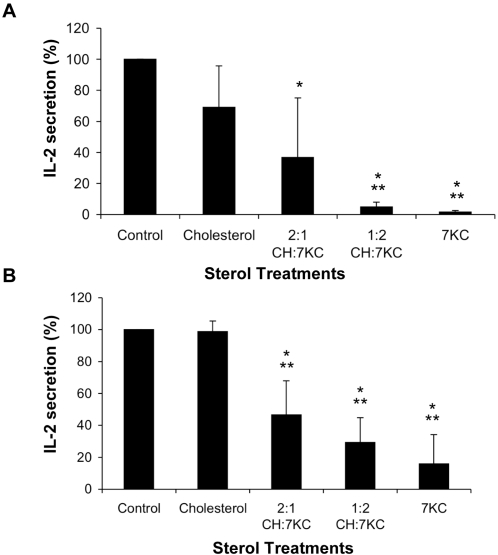
IL-2 secretion from stimulated sterol-enriched T cells. Jurkat 8.2 cells (A) and primary mouse T lymphocytes (B) were sterol treated with cholesterol and 7-ketocholesterol at the indicated ratios. Jurkat 8.2 cells were stimulated with antigen-conjugated B cells (A) and antigen exposed mouse T lymphocytes were activated with anti-CD3 mAb-coated beads (B). IL-2 secretion was determined by ELISA after 24h incubation. One and two asterisks indicates a significant difference to control cells of P<0.05 and P<0.001, respectively.

We next evaluated IL-2 secretion from CD3/CD28 expanded primary mouse lymphocytes by TCR stimulation with anti-CD3 antibody coated on 96-well plates (Fig. 8A). We also measured activation-induced IL-2 secretion of Jurkat 8.2 T lymphoma cells stimulated with MCC peptide-pulsed CH27 B cells (Fig. 8B). In both cell types, 7KC incorporation into the T cell membrane caused a dose dependent reduction of IL-2 secretion in response to T cell activation (P<0.05 at 2:1 CH:7KC and P<0.001 at 1:2 CH:7KC and 7KC alone). The Jurkat cells treated only with cholesterol secreted similar quantities of IL-2 as untreated controls. In primary T cells, IL-2 secretion was lower but not significantly reduced by the cholesterol-only treatment, which may be explained by the lower TCR surface expression under this condition. Similarly, to IL-2 secretion, phosphorylation of ERK1/2 after 24 h of stimulation was also decreased by 7KC enrichment in a dose-dependent manner (Fig. S5A). We further tested the responsiveness of sterol-enriched cells by assessing IL-2 transcription (Fig. S5B) when TCR activation is bypassed by stimulation with ionomycin and PMA. Only when cells are treated with 7KC alone did we find a reduced responsiveness in IL-2 transcription to non-specific activation.

Taken together, our data shows that 7KC incorporation specifically inhibits membrane condensation at T cell activation sites. This leads to reduced formation of LAT-based signaling complexes at plasma membrane and a failure of actin rings to polymerize at TCR activation sites. Consistently IL-2 secretion upon TCR engagement is reduced. These data indicate that T cell receptor signals are not effectively transduced when formation of ordered membrane domains is impaired.

## Discussion

The plasma membrane of T lymphocytes condenses at sites of T cell activation to form a zone rich in ordered membrane domains [17]. Rafts in lipid bilayers are defined as liquid-ordered (l_o_) phases in a liquid-disordered (l_d_) non-raft environment [33]. Thus membrane condensation at T cell activation sites is a physical reflection of raft accumulation. Here, we address the functional role of this membrane condensation in the activation of T lymphocytes. We found that enrichment of T cell membranes with 7-ketocholesterol (7KC) specifically inhibits membrane condensation at T cell activation sites. Following TCR activation 7KC–enriched cells exhibit normal early TCR signaling responses such as induction of calcium fluxes and phosphorylation of signaling proteins when whole cell lysates were examined. However, TCR triggering results in fewer TCR signaling complexes in the plasma membrane, reduced accumulation of tyrosine phosphorylated proteins on the cell surface and impaired re-structuring of actin cytoskeleton at the activation sites in 7KC-enriched T cells. Such differential localization of signaling activities correlates with an inhibition of the late response, IL-2 secretion following prolonged stimulation. Taken together, our data indicates that plasma membrane condensation is required for the sustained T cell activation process by possibly retaining signaling activities on the cell surface and that lipid-driven interactions are an important mediator of this condensation.

An increasing number of reports suggest a link between altered lipid homeostasis, as in hypercholesterolemia, and changes in immune cell activity [34] and immune responses. For example, failed T cell activation correlates with ganglioside expression in systemic lupus erythematosus [35], [36]. Cellular cholesterol levels are implicated in impaired T cell signaling [18] and in determining the internalization rates of surface receptors in anergic B cells [37]. Elucidating the molecular link between ordered membrane domains and immune cell activation could thus be important for the understanding of the effects of dyslipidemia on immune functions.

The functional importance of raft domains in T cell activation has been previously addressed by mβCD-mediated cholesterol extraction. Indeed, DRM-association of several membrane-anchored T cell signaling proteins, for example LAT and Src related tyrosine kinase Lck, is lost after cholesterol extraction [18]. However, mβCD treatment may also disturb other non-raft related functions [13]. This is exemplified in the case of T cell signaling by the inhibition of TCR triggering-induced Ca^2+^ fluxes by mβCD extraction, which is most likely due to the depletion of Ca^2+^ from intracellular stores [20] and thus independent of plasma membrane rafts. However 7KC loading had no effect on the induction of Ca^2+^ fluxes following triggering of TCR. Thus the machinery required for the induction of TCR-triggered Ca^2+^ fluxes remains functional upon inhibition of membrane condensation with 7KC. Furthermore, the general expression of T cell signaling proteins and tyrosine phosphorylation rates as well as cell viability are unaffected. This is strong evidence that the defective T cell activation responses caused by 7KC incorporation are a direct consequence of inhibition of membrane condensation at the activation sites.

Enrichment with 7KC effectively prevented the formation of ordered domains at T cell activation sites in a dose dependent manner. 7KC differs from cholesterol by an additional ketone group at the 7^th^ position of the sterol ring. The ketone group of 7KC protrudes perpendicular from the planar sterol ring and limits the depth of 7KC insertion into the membrane and its interaction with phospholipid acyl chains, orienting the two polar moieties of the oxysterol near the membrane-water interface [22], [38]. Importantly, the alignment of the sterol ring of 7KC with trans-configured saturated acyl chains of sphingo- and glycerolipids is impaired, causing decreased formation of ordered membrane domains [22]. The capability of an oxysterol to impair cell membrane condensation suggests that the physical properties of ordered domains in T cell membranes rely on the bilayer's sterol content and is thus lipid mediated.

Early TCR signals are transduced by TCR-LAT assemblies (TLAs) which form in the plasma membrane by anchorage of transmembrane linker protein LAT in the vicinity of triggered TCR [5], [30], [32]. In an elegant study, Douglass and Vale demonstrated that protein-protein interactions are the driving force for TLAs [39] creating cooperative association of cytoplasmic proteins with LAT clusters in the membrane [40], [41]. In response to TLAs, the membrane condenses at the T cell activation site [17]. We propose that protein-mediated, lateral clustering of LAT mediates plasma membrane condensation. LAT clustering and the anchoring of other raft-philic proteins at the T cell activation sites may drive the recruitment of pre-existing rafts or *de novo* formation of large raft domains. It is noteworthy that LAT recruitment and phosphorylation at the cell surface was more severely impaired by 7KC than signaling proteins further upstream of LAT. The balance of protein- and lipid-mediated interaction in the formation of TLAs remains to be investigated. Our data further suggests TCR triggering without membrane condensation results in insufficient signals to polymerize actin around the activation site. This in turn could affect the patterning of immunological synapses and thus the internalization and recycling rates of TCR signaling clusters. How membrane organization and actin restructuring are linked on the molecular level is an important subject of future research.

## Materials and Methods

### Cells and Reagents

Jurkat 8.2 cells, JCaM2 wtLAT cells, Jurkat cells and CH27 B cells were maintained as described previously [17], [42]. A1 (F) RAG-1^−/−^ CD4 T cells were isolated from spleen using Dynabeads Mouse CD4 (L3T4) and DETACHaBEAD Mouse CD4 (both Dynal Biotech) according to the manufacturers recommendations. Purified CD4 T cells were expanded using Mouse CD3/CD28 T cell expander (Dynal) in culture medium supplemented with 10 U/ml IL-2 for 6–8 days. Expanded cells were rested for 24h in IL-2 free medium before use. 7-ketocholesterol (7KC, 5-cholesten-3β-ol-7-one) was purchased from Steraloids (Newport, USA). Monoclonal antibodies KT3 (anti-mouse CD3) and OKT3 (anti-human CD3) were purified from hybridoma supernatant and purchased from eBioscience, respectively. Grb2 and ZAP-70 antibodies were purchased from Transduction Laboratories. PLCγ antibodies were from Cell Signaling Tech and its phospho-specific analogue from BD Biosciences. Phospho-specific ZAP70 and LAT antibodies were purchased from Cell Signaling Tech and Biosource, respectively. LAT rabbit antiserum was from Upstate Laboratories. Anti-β-actin (goat) was purchased from Abcam and anti-CD3γ (goat) from Santa Cruz.

### Sterol treatment

Aqueous stock solutions of 50 mg/mL methyl-ß-cyclodextrin (mßCD) complexed to 1.5 mg/mL sterol were prepared as described previously [43]. In brief, 5% mßCD in water was heated to 80°C and 4×10 μL aliquots of 15 mg/mL sterol in ethanol added every 5–10 min. 1–10×10^6^ cells were incubated with 15 μL (total) of mßCD-cholesterol, mßCD-7KC or a combination of the two sterols in 1mL RPMI supplemented with 1 mg/mL BSA and 50 mM HEPES for 30 min at 37°C. Cells were washed twice and conjugated, activated or lysed for sterol and gene analysis. Cellular sterols were extracted with hexane/methanol and analyzed on a reverse phase HPLC system as previously described [21].

### T cell activation

Jurkat, JCaM2 wt LAT or CD4 T cells were incubated with anti-CD3 antibody (OKT3)-coated M450 Dynabeads (Dynal) for isolation [30] or antibody-coated polystyrene beads [17] for microscopy on ice (bead to cell ratio 1:2) and activated for the indicated time at 37°C. Jurkat 8.2 cells (2 × 10^5^) were activated with B cell lymphoma cells CH27 (5×10^5^) in the presence or absence of moth cytochrome c (MCC) peptide 86–103 [42]. For microscopy CH27 were labeled with CMRA (CellTracker Orange, Invitrogen) [17].

### Laurdan microscopy

Verdi/Mira 900 multi-photon laser system and images were recorded simultaneously with emission in the range of 400–460nm and 470–530nm with a DM IRE2 Microscope (Leica, Australia) [24]. Microscopy calibrations were performed as described previously [23]. For other fluorescence and transmission images a helium-neon laser was used to excite CMRA (Ex: 543nm, Em: 550–620nm) and record transmission images (Ex: 633nm, Em: 650–720nm), respectively. For all images a 100× oil objective, N_A_ = 1.4 was used and imaged at RT.

### Image analysis

The Generalised Polarisation, GP, defined as
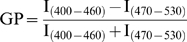
was calculated for each pixel using the two Laurdan intensity images, using the software WIT [24]. GP images were pseudo-colored in Adobe Photoshop. To determine GP values at activation sites or synapses, the mean GP area of the region of interest adjacent to the bead or APC was determined as previously described [17]. GP distributions were obtained from the histograms of the GP images, normalized (sum = 100) and fitted to two Gaussian distributions using the non-linear fitting algorithm (Microsoft Excel).

### TIRF microscopy

TIRF images were recorded with a Axiovert 200M microscope (Zeiss) and a 100× Plan-FLUAR objective (Zeiss), N_A_ = 1.4 as previously described [44]. Images were acquired with a 16-bit MicroMAX:512BFT CCD camera (Roper Scientific) driven by Metamorph (Universal Imaging). Exposure time for all images was 100 ms. Fluorescein isothiocyanate (FITC) and tetramethylrhodamine-isothiocyanate (TRITC) were excited with a multi-line Innova 900 laser (Coherent Scientific) at 488 nm coupled to a dual-port TIRF condenser (Till Photonics). Filter sets were from Chroma Technology Corp.

### Immunoisolation

Immunoisolations were performed as described previously [40]. Briefly, 5×10^7^ cells were incubated with antibody coated M500-subcellular beads (Dynal) for the indicated time at 37°C. The bead-cell conjugates were N_2_-cavitated using a nitrogen cavitation bomb (Model 4639, Parr Instrument Company), beads were retrieved with a magnet and subjected to immunoblotting analysis. Proteins or 30 μg of cell lysates were separated by 10% SDS-PAGE and transferred to Immobilon-P (Millipore) followed by incubation with primary antibodies and the appropriate peroxidase-conjugated secondary antibodies (Jackson Immunoresearch) and ECL detection (Amersham Pharmacia Biotech).

### Signaling

The signaling assay using Beadlyte 7-plex human TCR signaling kit (Millipore) were carried out as described by the manufacturer. Briefly, sterol-loaded and control Jurkat wt cells were activated with anti-CD3 UCHT1 antibody (Millipore) at 37°C for the indicated time periods. Cells were lysed and cell lysates incubated overnight with antibody-coated beads in 96-well, filter-bottomed plates. Beads were washed and incubated with the phospho-antibodies and detection reagents and analyzed with a Bio-Plex 200 System (BioRad) instrument.

### Calcium flux

5× 10^6^ untreated or sterol-loaded Jurkat T cells were incubated with 1 μM Indo-1 in 1ml RPMI (1% FCS) for 1h at 37°C in the dark. Subsequently cells were washed twice and re-suspended in 1ml RPMI. Cells were kept on ice in the dark and analyzed on a MoFlo (Cytomation) cell sorter at 37°C. The filter set-up for Indo-1 was for calcium bound Indo-1 violet FL-5 405/20 nm filter and unbound Indo-1 green FL-6 530/30 nm filter. Calcium flux was measured as a ratio between calcium bound Indo-1 and unbound or FL-5/FL-6. For localized stimuation, Fluo-4-labeled cells were incubated with anti-CD3-coated glass coverslips and fluorescence intensity recorded with an inverted microscope.

### IL-2 secretion

2.5×10^5^ Jurkat 8.2 cells were incubated together with 5×10^5^ CH 27 B cells in 0.5 mL RPMI containing 10% FCS and±2.5 μg/mL antigen for 18–24 hours. Cells were removed and the two aliquots of the supernatant (200 μL each) were used for human interleukin-2 (IL-2) ELISA assay (eBioscience). Background absorbance (determined from cells incubated without antigen) was subtracted. IL-2 standards were used to determine absolute IL-2 levels. For mouse IL-2 secretion, 96-well plates (Corning) were coated with 10 μg/ml anti-CD3 mAb (KT3) at 4°C overnight. Subsequently wells were washed three times with PBS and 2×10^5^ CD4 positive mouse T cells in 200 μl culture medium were plated on each well. 50 μl aliquots of the culture supernatant were used in a mouse IL-2 ELISA essay (eBioscience) after 24h incubation at 37°C.

### Cytotoxicity

10^6^ T cells (sterol-loaded or control) were incubated in cell culture medium for 24h at 37°C. The apoptosis and necrosis rate in the incubated cultures was determined using the Annexin-V-Fluos and propidium iodide staining kit (Roche Biosciences) according to the manufacturer's recommendations. Labeled samples were analyzed on a FACScalibur (BD Bioscience) fluorescence activated cell sorter. To determine caspase-3 activity, 2×10^6^ Jurkat wt cells (sterol-loaded and control) were lysed (10 mM Tris-HCl pH 7.4, 130 mM NaCl, 1% Triton X-100) and 15 μg of each lysate was incubated with 20 μM of Ac-DEVD-AMC (BD Biosciences) in protease assay buffer (40 mM HEPES pH 7.5, 20% glycerol and 4 mM DTT) for 1h at 37°C. Fluorescence emission upon Ac-DEVD-AMC cleavage was determined in a plate spectrofluorometer (Ex: 380 nm; Em: 445 nm). Lysates from Jurkat wt cells treated with 2 mM staurosporine (Sigma) for 4 h were used as a positive control. To determine ROS generation, 10^6^ Jurkat wt cells were pre-loaded with 10 μM DHR (Invitrogen) for 30 min before sterol enrichment. After 30 min incubation in culture media, the production of ROS was measured by fluorescence intensity of DHR by flow cytometry. To determine the mitochondrial membrane potential 10^6^ Jurkat cells were labeled with 5 μg of JC-1 (Invitrogen) and the red/green fluorescence shift analyzed by flow cytometry. The cellular ATP content of Jurkat was measured using the ATP bioluminescent assay kit from Sigma following the manufacturer instructions.

### CD3 surface expression

10^6^ T cells were incubated with anti-CD3-FITC antibody (eBioscience) for 1h at 4°C. The amount of surface fluorescence in the stained cells was determined by flow cytometry was analyzed using Weasel Flow Cytometry Software (WEHI, Melbourne).

### Gene transcription

Reverse transcription was performed with Superscript III (Invitrogen) using 1 μg total RNA in each reaction. qRT-PCR was performed on a RotorGene 3000 (Corbett Research) using Quantace SensiMix SyBr Green reagent as the detection system. β_2_ microglobulin was used as a housekeeping gene. Melting curve analysis was performed for each PCR product at the end of each run (confirming a single PCR product in each reaction).

### Statistics

Multiple comparisons were compared with one-way, nonparametric ANOVA with Tukey's post-testing.

## Supporting Information

Figure S1 FRET between tryptophan and Laurdan in activated T cells. Jurkat cells were labeled with Laurdan concentrations of 0–75 μM and conjugated to anti-CD3 mAb-coated beads (bead to cell ratio>1) for 10 min and fixed. A. 10^5^ cell-bead conjugates were resuspended in PBS in a quartz cuvette and tryptophan fluorescence (Ex = 280±5 nm, Em = 330±10 nm), Laurdan (Ex = 400±5 nm, Em = 450±10 nm) and FRET (Ex = 280±5 nm, Em = 450±10 nm) determined. FRET values were corrected for cross talk of Laurdan and tryptophan, which was determined in egg PC liposomes and activated T cells without Laurdan, respectively. Corrected FRET levels were normalized to Laurdan intensity (FRET^L^). The insert shows Laurdan intensity. B. GP values at the activation site was determined for Jurkat cells labeled with 2.5–75 μM Laurdan as described for Figure 2. Activation sites contain 4–5% of total Laurdan fluorescence. Means are indicated by horizontal lines and are: 0.436±0.065 (2.5 μM), 0.443±0.069 (5 μM), 0.442±0.058 (10 μM), 0.475±0.059 (25 μM), 0.484±0.078 (50 μM) and 0.485±0.059 (75 μM).
(0.32 MB TIF)

Figure S2 Effects of sterol enrichment on mitochondria potential (A), ATP levels (B), caspase-3 activity (C), ROS generation (D), protein (E) and gene expression (F). A. Percentage of cells with altered mitochondrial membrane potential (ΔΨ) as assessed by JC-1 staining and flow cytometry after sterol treatment. Cells were sterols loaded for 30 minutes or treated with 1mM H_2_O_2_ for 24 hours (positive control), and labeled with JC-1 for 20 minutes. Data are from 3 independent experiments. B. ATP content of Jurkat cells after sterol treatment. ATP levels of control and sterol-loaded Jurkat cells were measured using a luciferin/luciferase assay. Results are mean±SEM of four experiments. C. Caspase-3 activity was assessed by the cleavage of Ac-DEVD-AMC resulting in a fluorescent signaling. T cells treated with 2mM staurosporine for 4 h were used as a positive control (right y axis). 15 μg of lysate of sterol-enriched cells was incubated with 20 μM of Ac-DEVD-AMC for 1 h at 37°C. No difference in fluorescence intensity was found between control and sterol-enriched cells. D. Jurkat cells pre-labeled with 10 μM DHR (dihydrorhodamine 1,2,3) were either left untreated (control) or enriched in sterols. Treatment with 500mM H_2_O_2_ for 30 min at 37°C was used as a positive control. DHR fluorescence indicates the production of hydrogen peroxide (H_2_O_2_), hypochlorous acid (HOCl) and peroxynitrite anion (ONOO^−^). No significant differences between control and sterol treatments were found. E. Immunoblots of signaling proteins of whole cell lysates of wild-type Jurkat and Jurkat 8.2 cells. F. Relative mRNA levels of sterol-sensitive genes in Jurkat cells. Similar results were found in other types of T cells. A–D. One asterisk indicates a significant difference compared to control cells (P<0.05); two asterisks indicate a significant difference compared to cholesterol-enriched cells (P<0.05).
(1.09 MB TIF)

Figure S3 Immunoisolation of cholesterol-treated Jurkat cells and loading controls. A. Jurkat T cells (control or enriched in cholesterol only) were conjugated with magnetic beads coated with anti-CD3 monoclonal antibodies. Cell-bead conjugates were activated for 3–15 min at 37°C and subsequently homogenized by nitrogen cavitation at 4°C. Proteins from the recovered beads were separated by SDS page electrophoresis and probed for signaling proteins CD3ζ, ZAP70, LAT and PLCγ. Detection of proteins indicates recruitment to the activation site. Asterisk (*) denotes the antibody heavy chain. B. Quantification of heavy chain of the CD3 antibody conjugated to the beads in immuno-isolated membrane fragments indicates similar protein recovery from control, cholesterol- and 2:1 CH:7KC-enriched cells.
(0.51 MB TIF)

Figure S4 Fluorescence intensity of TIRF images. A–B. Maximum (hollow bars) and average fluorescence (filled bars) intensity of anti-phosphotyrosine staining (A) and phalloidin staining (B) were determined of 150–200 TIRF microscopy images per sterol treatment and normalized to control cells. Two asterisks indicate a significant difference to control cells of P<0.001. C–E. Integrated intensity of 150–200 TIRF images stained for ZAP70 phosphorylated at tryrosine 319 (C), LAT phosphorylated at tyrosine 191 (D) or PLCγ1 at tyrosine 783 (F). A–E. One asterisks indicates a significant difference compared to control cells (P<0.05); two asterisks indicate a significant difference compared to cholesterol-enriched cells (P<0.05).
(0.58 MB TIF)

Figure S5 Down-stream responses and responsiveness of sterol-enriched T cells. A. Phosphorylation of ERK1/2, expressed relative to total ERK1/2 in sterol-enriched T cells after 24 h activation with 5 μg of OKT3 (anti-CD3 antibody). B. IL-2 luciferase activity in sterol-enriched T cells treated with 1 μM ionomycin and 1 μM PMA for 24 h at 37°C. One asterisks indicates a significant difference compared to control cells (P<0.05); two asterisks indicate a significant difference compared to cholesterol-enriched cells (P<0.05).
(0.25 MB TIF)
