# Supplementary figures and images for: Functional Implications of Plasma Membrane Condensation for T Cell Activation
Source: PLoS One. 2008 May 28;3(5):e2262. doi: 10.1371/journal.pone.0002262 (PMC2384009; doi:10.1371/journal.pone.0002262)

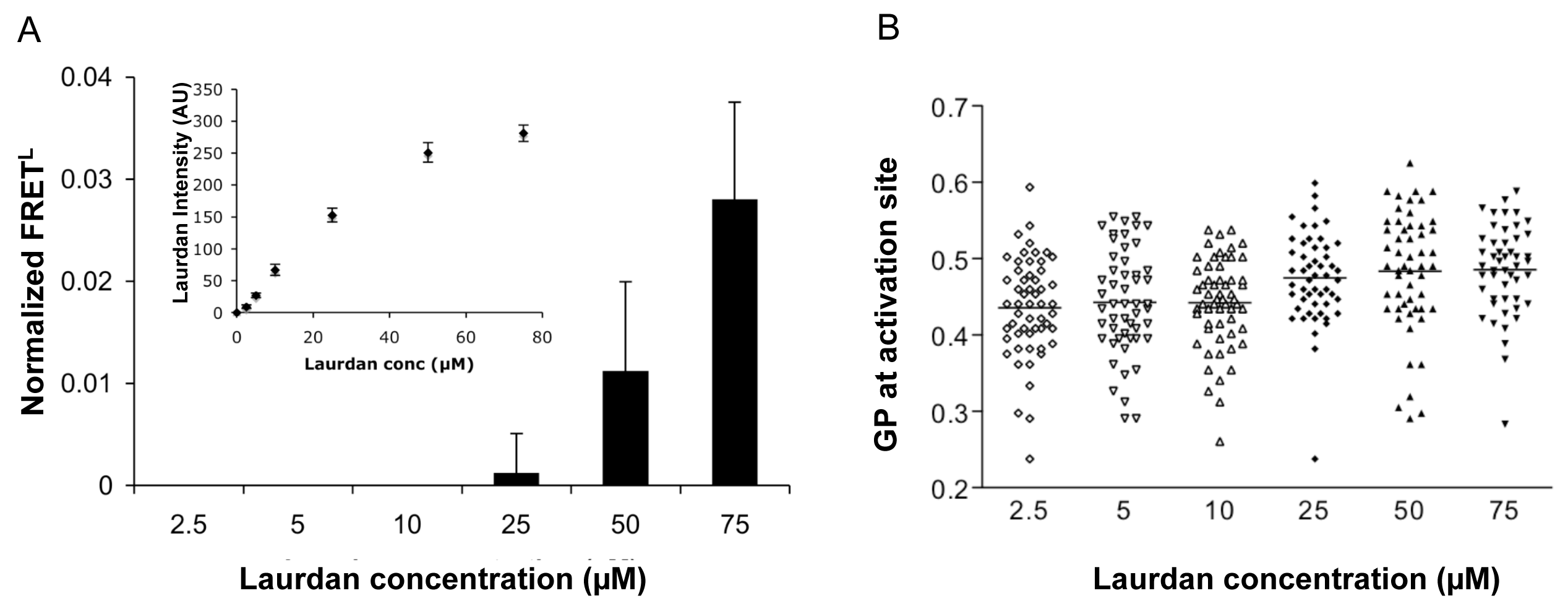

Supplement: Figure S1 — FRET between tryptophan and Laurdan in activated T cells. Jurkat cells were labeled with Laurdan concentrations of 0–75 μM and conjugated to anti-CD3 mAb-coated beads (bead to cell ratio>1) for 10 min and fixed. A. 105 cell-bead conjugates were resuspended in PBS in a quartz cuvette and tryptophan fluorescence (Ex = 280±5 nm, Em = 330±10 nm), Laurdan (Ex = 400±5 nm, Em = 450±10 nm) and FRET (Ex = 280±5 nm, Em = 450±10 nm) determined. FRET values were corrected for cross talk of Laurdan and tryptophan, which was determined in egg PC liposomes and activated T cells without Laurdan, respectively. Corrected FRET levels were normalized to Laurdan intensity (FRETL). The insert shows Laurdan intensity. B. GP values at the activation site was determined for Jurkat cells labeled with 2.5–75 μM Laurdan as described for Figure 2. Activation sites contain 4–5% of total Laurdan fluorescence. Means are indicated by horizontal lines and are: 0.436±0.065 (2.5 μM), 0.443±0.069 (5 μM), 0.442±0.058 (10 μM), 0.475±0.059 (25 μM), 0.484±0.078 (50 μM) and 0.485±0.059 (75 μM). (0.32 MB TIF) [file pone.0002262.s001.tif]

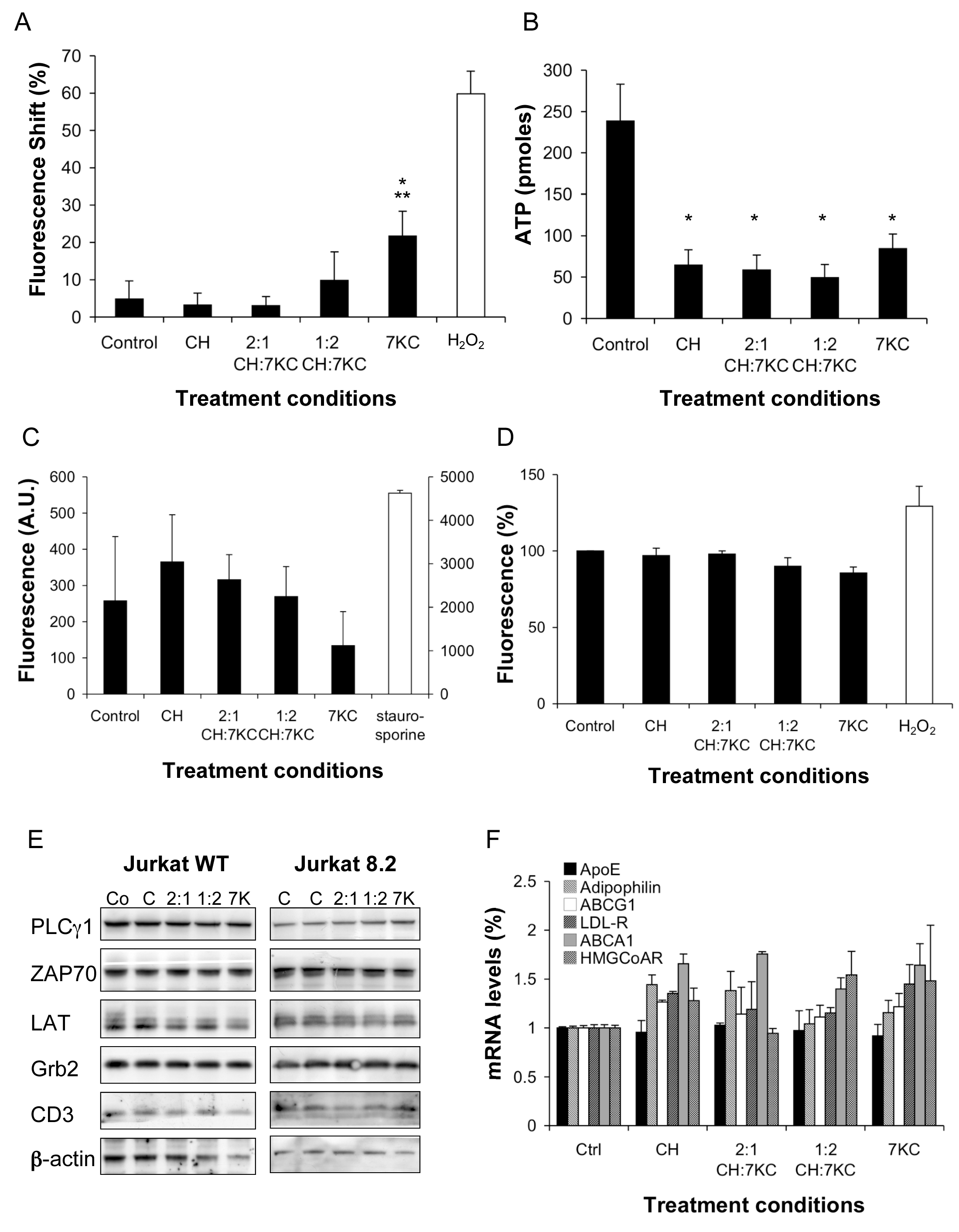

Supplement: Figure S2 — Effects of sterol enrichment on mitochondria potential (A), ATP levels (B), caspase-3 activity (C), ROS generation (D), protein (E) and gene expression (F). A. Percentage of cells with altered mitochondrial membrane potential (ΔΨ) as assessed by JC-1 staining and flow cytometry after sterol treatment. Cells were sterols loaded for 30 minutes or treated with 1mM H2O2 for 24 hours (positive control), and labeled with JC-1 for 20 minutes. Data are from 3 independent experiments. B. ATP content of Jurkat cells after sterol treatment. ATP levels of control and sterol-loaded Jurkat cells were measured using a luciferin/luciferase assay. Results are mean±SEM of four experiments. C. Caspase-3 activity was assessed by the cleavage of Ac-DEVD-AMC resulting in a fluorescent signaling. T cells treated with 2mM staurosporine for 4 h were used as a positive control (right y axis). 15 μg of lysate of sterol-enriched cells was incubated with 20 μM of Ac-DEVD-AMC for 1 h at 37°C. No difference in fluorescence intensity was found between control and sterol-enriched cells. D. Jurkat cells pre-labeled with 10 μM DHR (dihydrorhodamine 1,2,3) were either left untreated (control) or enriched in sterols. Treatment with 500mM H2O2 for 30 min at 37°C was used as a positive control. DHR fluorescence indicates the production of hydrogen peroxide (H2O2), hypochlorous acid (HOCl) and peroxynitrite anion (ONOO−). No significant differences between control and sterol treatments were found. E. Immunoblots of signaling proteins of whole cell lysates of wild-type Jurkat and Jurkat 8.2 cells. F. Relative mRNA levels of sterol-sensitive genes in Jurkat cells. Similar results were found in other types of T cells. A–D. One asterisk indicates a significant difference compared to control cells (P<0.05); two asterisks indicate a significant difference compared to cholesterol-enriched cells (P<0.05). (1.09 MB TIF) [file pone.0002262.s002.tif]

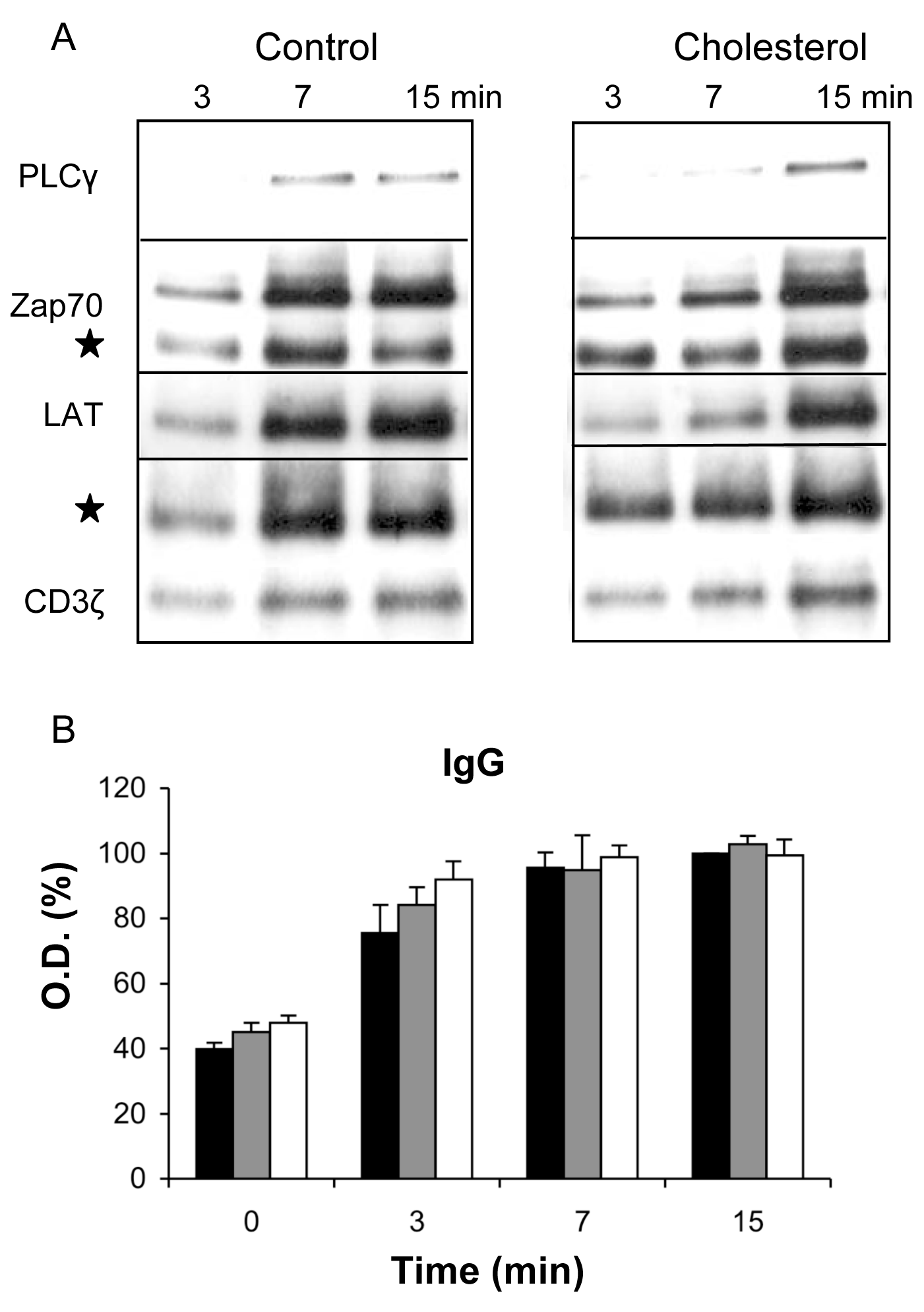

Supplement: Figure S3 — Immunoisolation of cholesterol-treated Jurkat cells and loading controls. A. Jurkat T cells (control or enriched in cholesterol only) were conjugated with magnetic beads coated with anti-CD3 monoclonal antibodies. Cell-bead conjugates were activated for 3–15 min at 37°C and subsequently homogenized by nitrogen cavitation at 4°C. Proteins from the recovered beads were separated by SDS page electrophoresis and probed for signaling proteins CD3ζ, ZAP70, LAT and PLCγ. Detection of proteins indicates recruitment to the activation site. Asterisk (*) denotes the antibody heavy chain. B. Quantification of heavy chain of the CD3 antibody conjugated to the beads in immuno-isolated membrane fragments indicates similar protein recovery from control, cholesterol- and 2:1 CH:7KC-enriched cells. (0.51 MB TIF) [file pone.0002262.s003.tif]

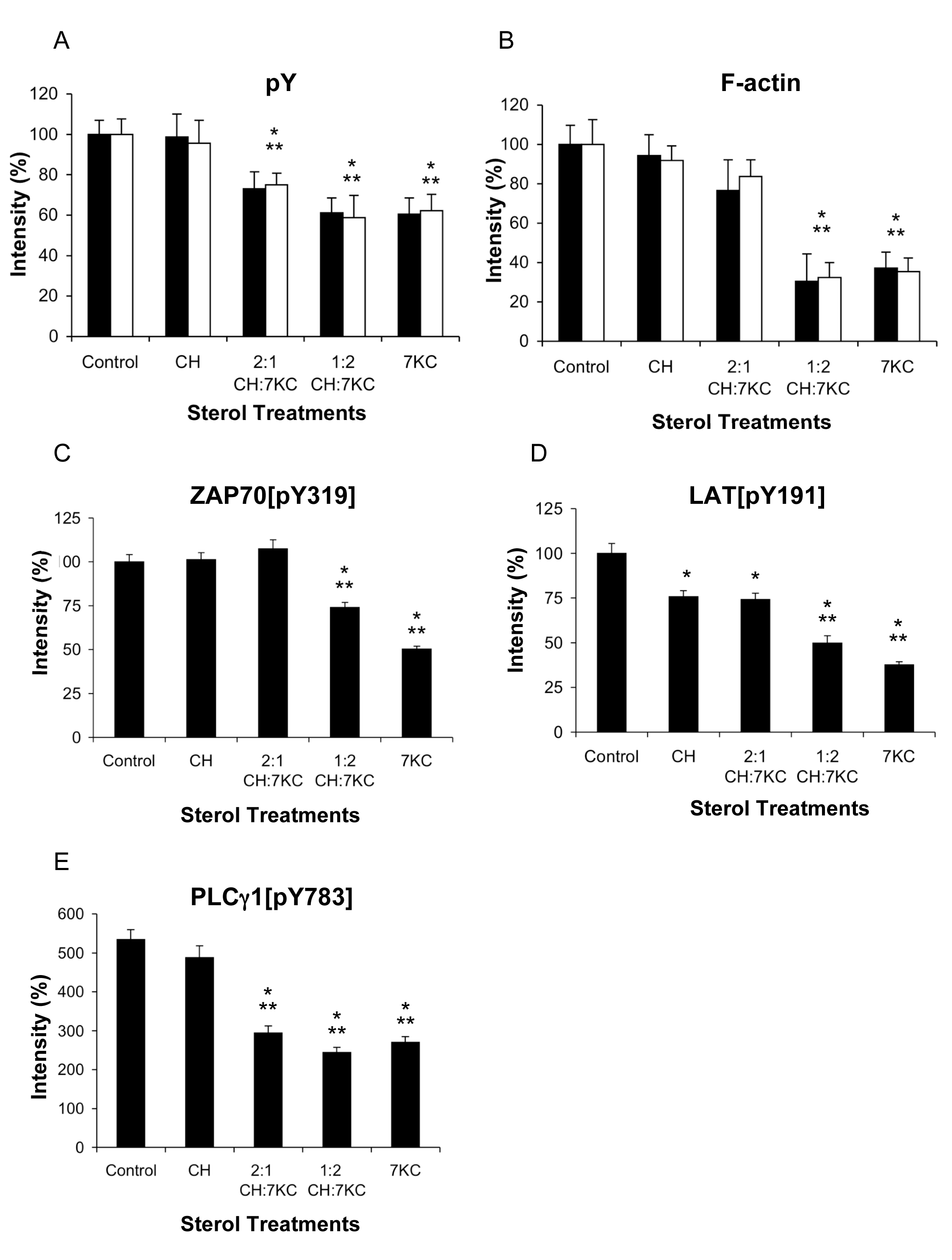

Supplement: Figure S4 — Fluorescence intensity of TIRF images. A–B. Maximum (hollow bars) and average fluorescence (filled bars) intensity of anti-phosphotyrosine staining (A) and phalloidin staining (B) were determined of 150–200 TIRF microscopy images per sterol treatment and normalized to control cells. Two asterisks indicate a significant difference to control cells of P<0.001. C–E. Integrated intensity of 150–200 TIRF images stained for ZAP70 phosphorylated at tryrosine 319 (C), LAT phosphorylated at tyrosine 191 (D) or PLCγ1 at tyrosine 783 (F). A–E. One asterisks indicates a significant difference compared to control cells (P<0.05); two asterisks indicate a significant difference compared to cholesterol-enriched cells (P<0.05). (0.58 MB TIF) [file pone.0002262.s004.tif]

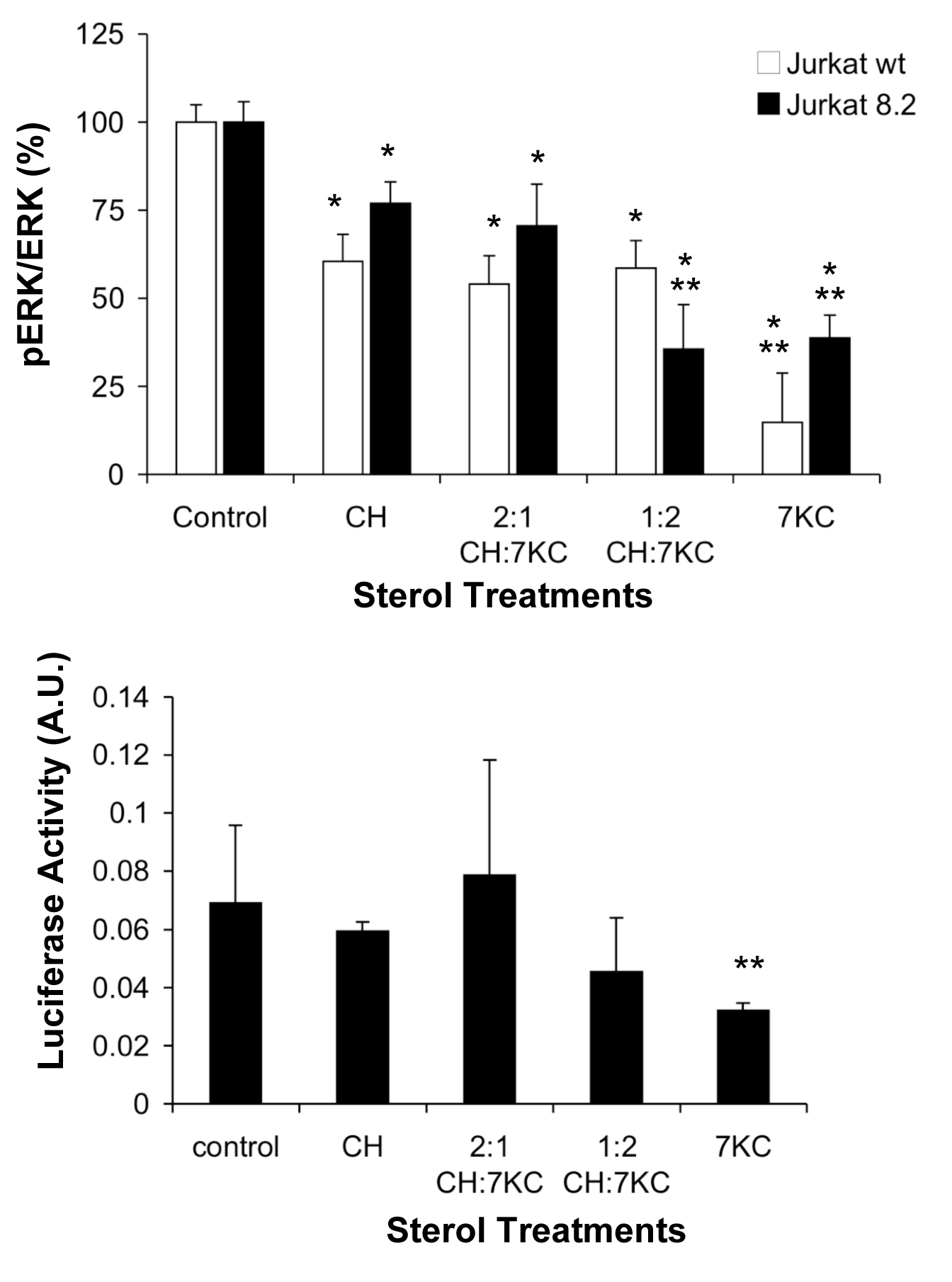

Supplement: Figure S5 — Down-stream responses and responsiveness of sterol-enriched T cells. A. Phosphorylation of ERK1/2, expressed relative to total ERK1/2 in sterol-enriched T cells after 24 h activation with 5 μg of OKT3 (anti-CD3 antibody). B. IL-2 luciferase activity in sterol-enriched T cells treated with 1 μM ionomycin and 1 μM PMA for 24 h at 37°C. One asterisks indicates a significant difference compared to control cells (P<0.05); two asterisks indicate a significant difference compared to cholesterol-enriched cells (P<0.05). (0.25 MB TIF) [file pone.0002262.s005.tif]
